# Effects of Virtual Reality Based on Fall Prevention Intervention: A Systematic Review and Meta-Analysis

**DOI:** 10.3390/healthcare13151845

**Published:** 2025-07-29

**Authors:** Bom-Mi Park, Heejung Choi, Harim Jeong

**Affiliations:** 1 Department of Nursing, Research Institute (RIBHS), College of Biomedical & Health Science, Konkuk University, Chungju-si 27478, Republic of Korea; 2 Department of Nursing, Chungcheong University, Cheongju-si 28171, Republic of Korea

**Keywords:** virtual reality, fall prevention, fall efficacy scale, number of falls, systematic review

## Abstract

**Background/Objectives**: Falls are recognized as a leading cause of injury, with approximately one in ten incidents resulting in physical injury. Although virtual reality (VR)-based interventions have been explored for fall prevention, systematic reviews and meta-analyses remain limited. This study aimed to assess research trends and evaluate the effectiveness of VR-based fall prevention through a systematic review and meta-analysis. **Methods**: This review was conducted following the Preferred Reporting Items for Systematic Reviews and Meta-Analyses (PRISMA) guidelines. A comprehensive literature search was carried out in PubMed, EBMASE, Cumulative Index to Nursing and Allied Health Literature (CINAHL), Cochrane Library, and Korean databases from their inception through 31 December 2024. A total of 49 studies met the inclusion criteria, and a meta-analysis was conducted on 37 studies with available data using “R” 4.4.1 software. Effect sizes (ESs) and 95% confidence intervals (CIs) were calculated for key outcomes. **Results**: The VR-based interventions showed a statistically significant positive effect on falls self-efficacy, as measured by the Falls Efficacy Scale (FES) (ES = 0.28, 95% CI: 0.17–0.39, *p* < 0.001). However, no significant reduction was observed in the number of falls (ES = −0.31, 95% CI: −0.80–0.17, *p* = 0.20). Subgroup analysis by participant medical condition for the FES revealed the largest effects in the Parkinson’s disease (PD) group (ES = 0.61), followed by the multiple sclerosis (MS) (ES = 0.34), the “other” group (ES = 0.25), and “healthy” participants (ES = 0.24). A statistically significant reduction in the number of falls was observed only in the MS group (ES = −0.56). **Conclusions**: VR-based interventions are effective in improving falls self-efficacy, particularly among individuals with neurological conditions, such as Parkinson’s disease and multiple sclerosis. However, evidence for a reduction in actual fall incidence remains limited. Further large-scale, long-term studies are needed to evaluate the sustained impact of VR interventions on fall prevention outcomes.

## 1. Introduction

Falls are the most common cause of injury among adults aged 65 and older, with one out of every ten falls resulting in an injury. Approximately 3 million emergency department visits occur annually because of falls in the elderly, and approximately 319,000 older adults are hospitalized each year because of hip fractures [1]. Additionally, falls frequently occur in patients with various conditions, such as Parkinson’s disease, multiple sclerosis, and stroke [2]. Falls cause significant injuries, such as dislocations or fractures, often resulting in hospitalization for moderate-to-severe injuries. Furthermore, prolonged hospitalization can lead to secondary complications, such as pressure ulcers and delirium, which can result in death [3]. Even if death does not occur, many individuals are unable to engage in activities on their own after discharge or are readmitted for institutional care rather than returning home [4].

Consequently, the importance of balance exercises for fall prevention has recently been emphasized, and methods for balance exercises that are easy and simple to perform independently have been introduced [5]. Moreover, virtual reality (VR) technology is widely used in the medical field [6], providing environments that are difficult to experience in real life and allowing interaction with the surrounding environment without direct experience [7].

VR is a form of digital therapy that integrates multiple stimuli through the visual, auditory, tactile, and proprioceptive systems, providing an opportunity for practice in a realistic environment resembling actual objects and events [2]. It has also been reported to contribute to improvements in balance and motor function [8]. In particular, multisensory activation through VR-based interventions is known to involve the mirror neuron system, thereby promoting neuroplasticity in intact cortical regions, which may contribute to the recovery and substitution of lost functions [9]. In addition, visual feedback is frequently utilized in VR interventions, inducing sensory illusions during active movement execution. This sensory feedback facilitates the reorganization of sensorimotor circuits, leading to improvements in postural stability and the motor skills required for maintaining dynamic balance [10,11]. Despite these physiological advantages, studies involving individuals with neurological disorders commonly have the limitation of small sample sizes, and there is a lack of research evaluating specific outcome variables, such as balance confidence, fear of falling, and postural control [11].

Recent studies have explored not only traditional fall prevention programs, such as fall prevention [12], Otago exercise [13], and self-management [14], but also the use of VR technology in rehabilitation and exercise therapy. In particular, VR technologies, such as the C-Mill (treadmill), Nintendo Wii, and Microsoft Xbox (Kinect)-based gait and balance training, have been actively investigated for their effects on various fall-related variables, including the Falls Efficacy Scale (FES), number of falls, Fall Risk Index (FRI), Fall Risk Assessment Tool (FRAT), Berg Balance Scale (BBS), and Timed Up and Go (TUG) [15,16,17,18]. The outcome measures used in these studies can be broadly categorized into two types. First, subjective efficacy refers to an individual’s perceived confidence in performing daily activities. Second, clinical efficacy pertains to objectively measurable outcomes related to physical function, such as actual fall events, the Berg Balance Scale (BBS), and the Timed Up and Go (TUG) test [15,16,17].

Previous systematic reviews have reported that VR-based fall prevention interventions are effective in improving balance control and reducing fall risk across various populations [2,11]. Although substantial research has investigated the effects of VR interventions on the FES and the number of falls, FRI, and FRAT [15,16,17,18], comprehensive syntheses through systematic reviews and meta-analyses are still insufficient. Therefore, this study aims to systematically analyze the effectiveness of VR-based interventions in fall prevention, with a specific focus on primary outcomes, such as the FES and the number of falls.

Moreover, despite the growing number of studies targeting specific disease populations, such as dizziness [18], stroke [19], Parkinson’s disease (PD) [20], and multiple sclerosis (MS) [21], comparative analyses of effect sizes across these disease groups remain insufficient. Accordingly, this study also seeks to explore differences in intervention effectiveness by disease type, thereby evaluating the applicability of VR-based fall prevention interventions in populations with specific medical conditions.

Based on this background, the present study aims to conduct a comprehensive review of recent domestic and international studies on VR-based fall prevention interventions, evaluate their effects across key outcome variables and target populations, and propose effective VR intervention strategies applicable to clinical nursing practice.

In our study, two research questions are presented. Research question 1: What is the effectiveness of the VR-based fall prevention intervention program? Research question 2: Does the effectiveness of the program differ according to the participants’ disease groups?

## 2. Materials and Methods

### 2.1. Study Design

We conducted a systematic review and meta-analysis of international and Korean studies on the effectiveness of VR-based fall prevention programs using the Preferred Reporting Items for Systematic Reviews and Meta-Analyses (PRISMA) reporting guideline [22]. The protocol was registered with the International Platform of Registered Systematic Review and Meta-Analysis Protocols (INPLASY, registration number: 202560045) and was assigned the DOI 10.37766/inplasy2025.6.0045.

### 2.2. Inclusion and Exclusion Criteria

(1) For the inclusion criteria, the core data selected were the participants, interventions, comparisons, outcomes, and study design (PICO-SD).
  - Population: This study included all Korean and international participants.
  - Intervention: The intervention was fall prevention utilizing virtual reality (VR).
  - Comparison: The control groups received either no intervention or maintenance of the existing program.
  - Outcome: The intervention outcome was a study that presented fall-related variables, including falls.
  - Study design: Randomized and non-randomized controlled trials were included.
(2) The exclusion criteria were as follows: a. studies for which the original text was unavailable; b. studies published in a language other than English or Korean; and c. publications written in the form of qualitative studies, reviews, dissertations, and conference presentation abstracts.

### 2.3. Search Strategy and Selection

Three investigators conducted a literature search of VR-based fall prevention interventions and selected research papers published in Korean and international journals from their inception to December 2024. In cases of disagreement, the researchers engaged in thorough discussions and continued deliberating until a unanimous consensus was achieved.

The literature searches were conducted in PubMed, EMBASE, Cumulative Index to Nursing and Allied Health Literature (CINAHL), Cochrane Library, and Korean databases (Research Information Sharing Service (RISS), Korean Studies Information Service System (KISS), Database Periodical Information Academic (DBpia), and KoreaMed). The search terms VR or virtual realt*, accidental falls, fall, or fall prevention were used. Boolean operators OR and AND were applied (Supplementary Table S1).

**Figure 1:**
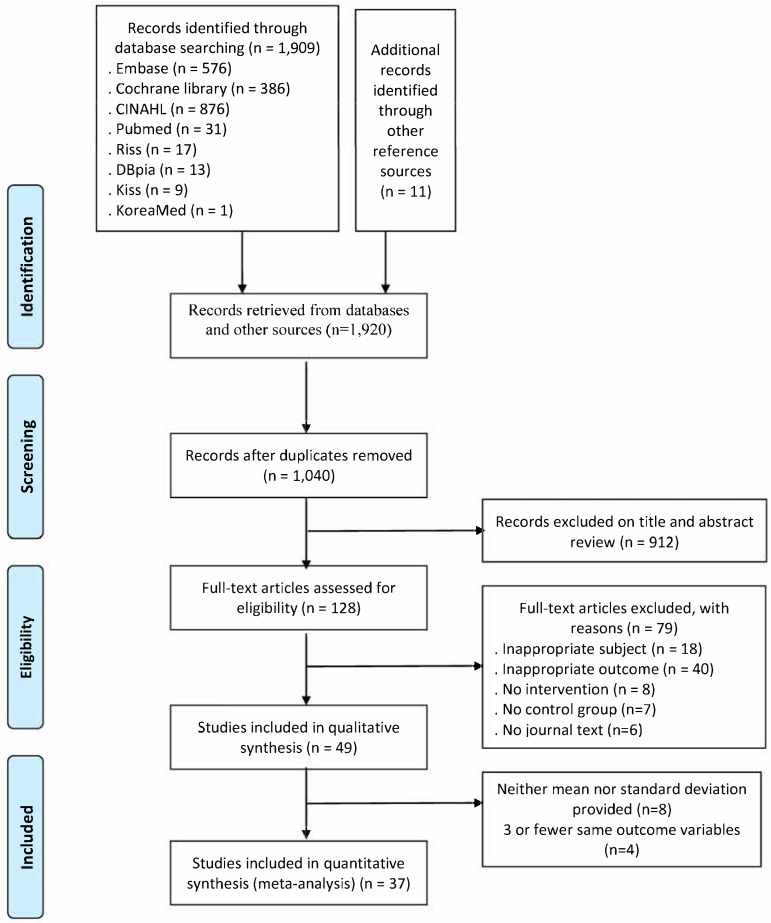
Study selection flow diagram.

Through the literature searches, 1869 international and 40 domestic papers were selected, totaling 1909 papers. Eleven papers were manually searched. After removing duplicates, the abstracts and titles of 1040 papers (1.020 international and 20 domestic) were reviewed, and 128 papers were selected in the first stage. In the second selection process, after reviewing the full texts, 18 papers with inappropriate subjects, 40 papers without outcome variables, 8 non-intervention studies, 7 papers without control groups, and 6 papers without original texts were excluded, leaving a total of 49 papers selected for this systematic literature review. After excluding eight papers in which the means and standard deviations of the outcome variables could not be calculated and four papers with fewer than three of the same variables, a final set of thirty-seven papers was selected for the meta-analysis (Figure 1).

### 2.4. Risk of Bias

The internal validity of the included randomized controlled trials (RCTs) and non-randomized controlled trials (NRCTs) was assessed using Cochrane’s Risk of Bias 2.0 (RoB 2.0) and the Risk of Bias in Non-randomized Studies of Interventions (RoBINS-I). RoB 2.0 evaluates bias based on the randomization process, deviations from intended interventions, missing outcome data, outcome measurement methods, and the selection of reported results. The risk of bias for each item was categorized as “low”, “some concern”, or “high” [23]. In contrast, RoBINS-I evaluates bias based on confounding factors in a study, selection bias in participant inclusion, bias in the classification of interventions, bias due to deviations from intended interventions, bias resulting from missing data, bias in the measurement of outcomes, and bias in the selection of reported results. The risk of bias for each item was categorized as “low”, “moderate”, or “serious” [24]. The risk of bias for the items was marked as low, moderate, serious, critical, or no information. Three researchers independently assessed the studies, and any discrepancies were reviewed until a consensus was reached to draw conclusions.

### 2.5. Assessment of Evidence Quality

The certainty of evidence was assessed using the Grading of Recommendations, Assessment, Development, and Evaluation (GRADE) system, which allows for the evaluation of the following five domains: (i) risk of bias, (ii) inconsistency, (iii) indirectness, (iv) imprecision, and (v) overall quality of evidence.

### 2.6. Statistical Analysis

Data collection for this systematic literature review and meta-analysis was conducted by three researchers, who coded author (year), design, country, age, disease, sample size, min, frequency, duration, follow-up, technology, intervention, control, and outcome. The collected data were organized and collated using the EndNote X9 and Excel 2024 programs.

#### 2.6.1. Effect Size Analysis

The effect size and publication bias were calculated using the meta-analysis packages “meta” and “metafor” of the R program.

The effect size was calculated using the mean and standard deviation of the fall variable or the suggested effect size. The analysis was conducted immediately after the intervention and during the follow-up period.

According to the criteria of Cohen [25], if the effect size was less than 0.20, it was interpreted as a small effect size; if it was approximately 0.50, it was interpreted as a medium effect size; and if it was 0.80 or more, it was interpreted as a large effect size, and the 95% confidence interval was applied to verify statistical significance.

A random effects model was used to calculate the effect size in the meta-analysis. A fixed-effects meta-analysis assumes that all studies estimate the same treatment effect; however, a random-effects meta-analysis allows for differences in treatment effects across studies [26]. Therefore, a random-effects meta-analysis was performed. According to the criteria of Higgins and Thompson [27], the quantification of heterogeneity was judged by the adjectives low, medium, and high, with I^2^ values of 25%, 50%, and 75%, respectively.

#### 2.6.2. Publication Bias and Sensitivity Analysis

After reviewing the data, a publication bias analysis was conducted to check for errors and consider their impact [28]. Publication bias was visually assessed using a funnel plot, and asymmetry was evaluated. Additionally, for the statistical analysis of publication bias, Egger’s regression intercept and the trim-and-fill method, developed by Duval and Tweedie [29], were used to assess the extent to which publication bias might have affected the study results.

In addition, a sensitivity analysis was performed to assess the robustness of the findings, using visual tools such as Baujat plots and influence plots.

### 2.7. Ethical Considerations

This study was exempt from review by the Institutional Review Board of Konkuk University (IRB No. 7001355-202406-E-808), as it involved the analysis of previously published results.

## 3. Results

### 3.1. Study Characteristics

A total of 49 studies were included in this systematic literature review, of which 44 were RCTs. The sample sizes varied considerably, ranging from 16 participants [30] to a maximum of 630 participants [31]. Smaller sample sizes predominated, where 18 studies enrolled 30 or fewer participants (N ≤ 30), whereas only five studies included 100 or more participants (N ≥ 100). Follow-up assessments were conducted in 22 studies, with follow-up durations ranging from 4 weeks to 1 year after intervention completion. Specifically, follow-ups at 6 months were reported in seven studies, at 3 months (or 12 weeks) in six studies, and at 1 year in three studies.

The Nintendo Wii was the most frequently used VR technology for the interventions and was applied in 11 studies. Treadmill-based VR systems, such as V-time and C-Mill, were utilized in three studies each, totaling six studies. Other VR technologies included Xbox Kinect and balance rehabilitation units, although two studies [32,33] did not specify the type of technology used. An analysis of exercise components in the interventions revealed that many studies focused primarily on balance training. This was followed by interventions described as VR exercise, Exergames, rehabilitation, or therapy, which were designed to achieve combined effects. The next most common type of intervention involved gait-focused training [18,19,20,33,34,35,36,37], often incorporating the use of a treadmill. In other studies [15,16,17,38,39,40,41,42], a combination of various conventional exercise and VR-based programs was employed as an intervention. VR technology was integrated into the Otago Exercise Program [43], as well-established fall prevention.

The number of intervention sessions, calculated by multiplying weekly frequency by intervention duration, ranged from 8 to 36 sessions. Most studies administered 10 or more sessions, with 12 and 18 sessions being the most common. The control groups typically received conventional (usual) care or exercises similar to the experimental interventions without VR technology. Three studies provided only educational or informational brochures to the control participants. Additionally, two studies included more than one control group, and eight studies implemented no intervention in the control group.

Fall-related efficacy was assessed in 36 studies using the Falls Efficacy Scale (FES), a self-report questionnaire, and its variants (all self-report), including the FES-International (FES-I), the Modified FES (MFES), and the Short-Form FES-I. The FES-I was the most commonly employed, which was used in 22 studies. Only 13 studies included the number of falls as an outcome variable, indicating a limited focus on actual fall incidence. Among them, eight conducted follow-up assessments, underscoring the need for prolonged observation to accurately evaluate intervention effects on falls. Three studies [44,45,46] reported that the number of falls was assessed by healthcare professionals, while the remaining studies relied on the participants’ self-reports. In particular, follow-up assessments frequently depend on participants’ recall of fall incidents.

Regarding participant health status, 20 studies did not specify any particular medical condition, and these participants were categorized as “healthy” for this review. The mean age of the participants in the studies involving “healthy” participants was consistently 65 years or older. Among the studies specifying health conditions, eight involved patients with Parkinson’s disease (PD), five with multiple sclerosis (MS), and four with stroke. Similar to the healthy participants, the mean age in the PD studies was 65 years or older. Conversely, the participants in the five studies involving patients with MS had a mean age in their 30s or 40s, reflecting the typical onset age of MS, which ranges from 20 to 40 years. For stroke patients, only one out of four studies reported an average age of 65 or above; the remaining studies included participants in their early 60s or 50s. Additionally, in this review, studies targeting a specific health condition but limited to two or fewer studies, as well as those involving participants with mixed health conditions, were classified as “other”. Among these studies, one or two studies targeted participants with specific health issues, such as diabetes mellitus (DM), mild cognitive impairment (MCI), fibromyalgia, osteoarthritis, osteoporosis, dizziness, and balance disorders. Notably, only the study involving patients with fibromyalgia reported a mean participant age in the 50s, consistent with the typical onset age of fibromyalgia; the participants in the other condition-specific studies were predominantly aged 65 years or older. Furthermore, two studies included participants from three distinct groups with different diseases, including elderly and PD patients, all with an average age above 65 years. Finally, the mean age of the study participants appeared to be closely related to the specific health conditions addressed (Table 1).

#### 3.1.1. Results of Risk of Bias

**Figure 2:**
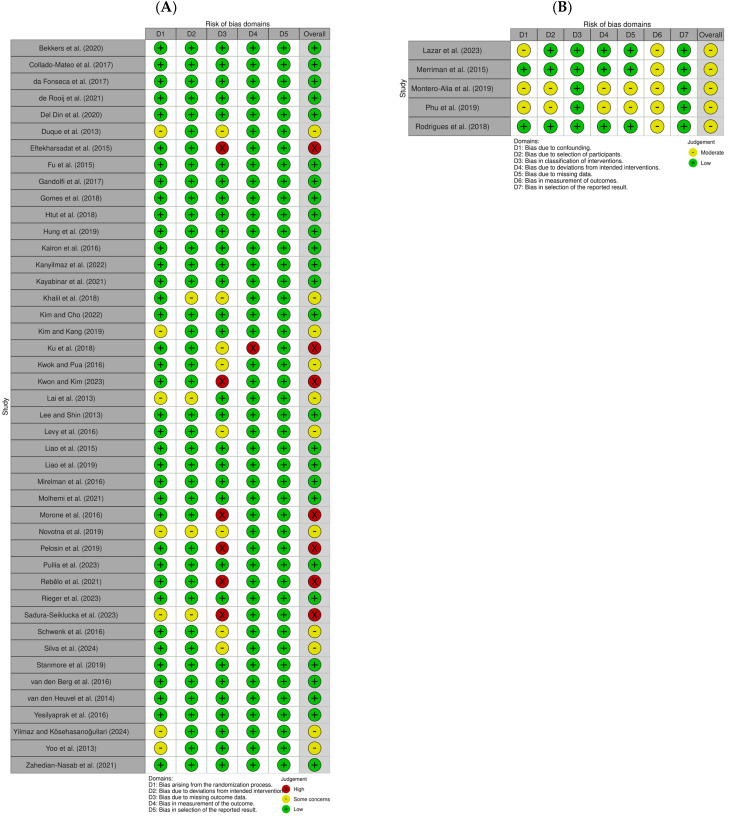
Risk of bias assessment of the included studies. (**A**) Randomized studies; (**B**) non-randomized studies [15,16,17,18,19,20,21,30,31,32,33,34,35,36,37,38,39,40,41,42,43,44,45,46,47,48,49,50,51,52,53,54,55,56,57,58,59,60,61,62,63,64,65,66,67,68,69,70,71].

A quality assessment of the selected papers was conducted by three researchers using RoB2 from the Cochrane Library for 44 papers and ROBINS-I for 5 papers. In the quality assessment of the RCTs, 26 papers [15,17,18,19,20,21,34,35,36,37,40,45,46,47,48,50,51,52,53,55,59,60,67,68,69,71] were classified as “low risk,” 11 papers [16,30,38,43,44,54,56,58,62,66,70] as “some concerns,” and 7 papers [33,39,41,42,49,57,64] as “high risk.” In the quality assessment of the NRCTs, five papers [31,32,61,63,65] were rated as “moderate,” and none were rated as “low” (Figure 2).

#### 3.1.2. Certainty of Evidence: GRADE

The GRADE system was applied to assess the certainty of evidence for the FES (36 studies) and the number of falls (13 studies). As a result, the certainty of evidence was rated as “moderate” for the FES and “very low” for the number of falls (Supplementary Table S2).

### 3.2. Meta-Analysis

Of the 49 studies, 37 were included in the meta-analysis. The studies that provided means and standard deviations or those for which calculations could be made mathematically, even without these values, were integrated and analyzed using metagen. However, eight studies for which the effect sizes could not be calculated and four studies with fewer than three outcome variables (FRI, FoF, and number of fallers) were excluded from the meta-analysis. Therefore, the meta-analysis included studies that assessed the FES and the number of falls.

#### 3.2.1. Effects of VR-Based Fall Prevention Intervention on the FES and the Number of Falls

The effect sizes of VR-based fall prevention interventions were analyzed in 37 studies in which an effect size analysis was possible. The effects of using a VR fall prevention program in terms of the FES (k = 46) and the number of falls (k = 13) are shown.

Among the tools presented in this study, the FES-I, the short FES-I, and the number of falls showed negative effects, whereas the FES and MFES showed positive effects. Therefore, when calculating the effect size related to this study, the negative effect of the FES was analyzed with the adjusted sign. The FES, MFES, FES-I, and short FES-I scores were classified as the FES for the meta-analysis.

The effect size for the FES was 0.28 (CI: 0.17–0.39, *p* ≤ 0.001), and for the number of falls, it was −0.31 (CI: −0.80–0.17, *p* = 0.2), both of which were small effect sizes according to Cohen’s criteria [25].

#### 3.2.2. Subgroup Analysis

A subgroup analysis was conducted based on disease, number of interventions, and time immediately after follow-up.

For the FES, when categorized by disease, the significant effects were strongest in the PD group (ES = 0.61), followed by the MS group (ES = 0.34), the “other” group (ES = 0.25), and the “healthy” group (ES = 0.24). A significant effect was observed on the number of falls in the MS group (ES = −0.56).

**Figure 3:**
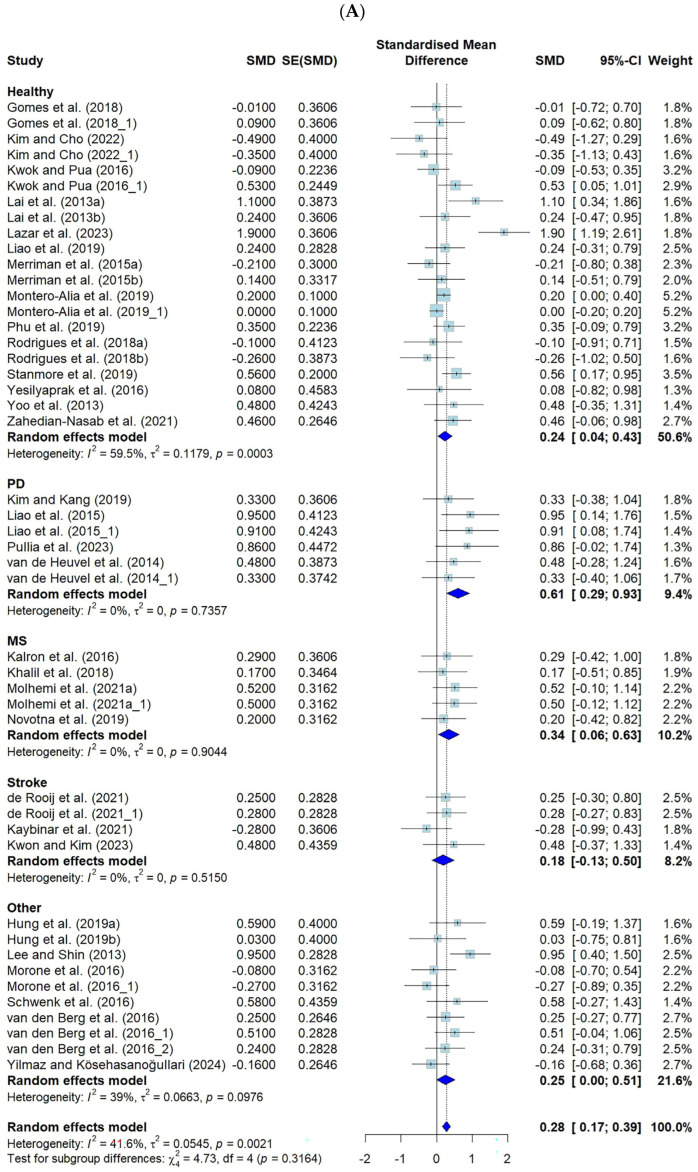

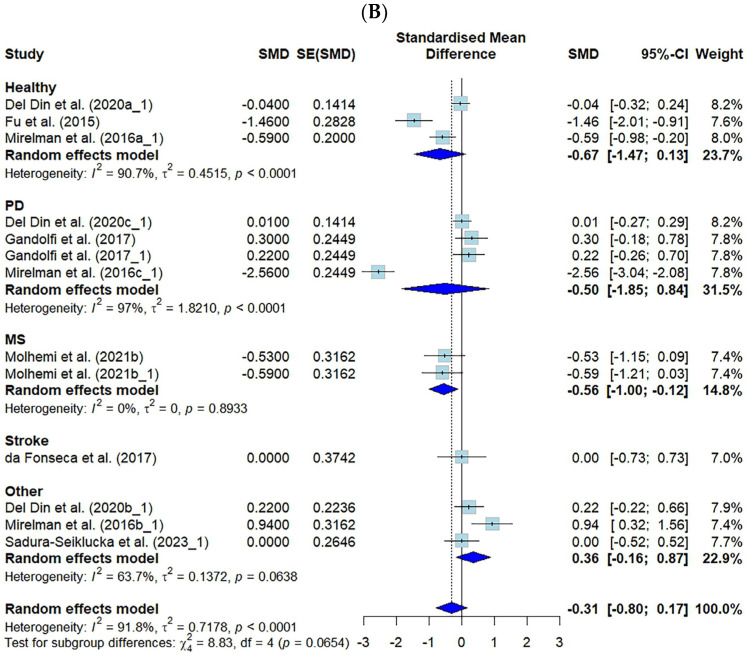
Forest plot of the effect of VR-based fall prevention intervention on the FES score and the number of falls. (**A**) Falls Efficacy Scale score. (**B**) Number of falls [15,17,19,21,31,32,34,35,36,38,39,40,41,42,43,45,46,48,50,52,53,54,55,56,58,59,60,61,62,63,65,66,67,68,69,70,71].

The FES showed moderate heterogeneity, at 41.6% (*p* = 0.002), and the number of falls showed high heterogeneity, at 91.8% (*p* < 0.001) (Figure 3).

**Figure 4:**
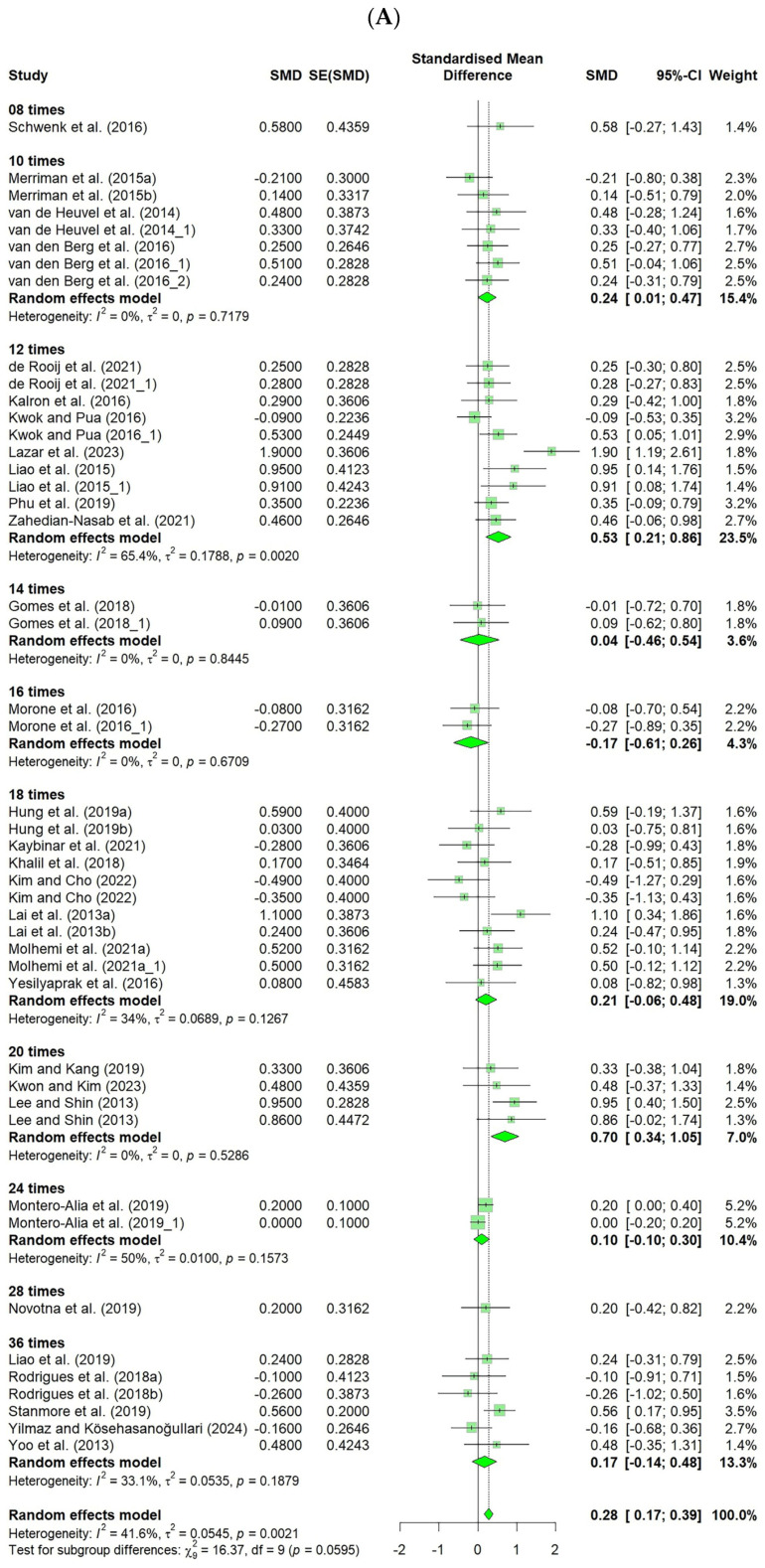

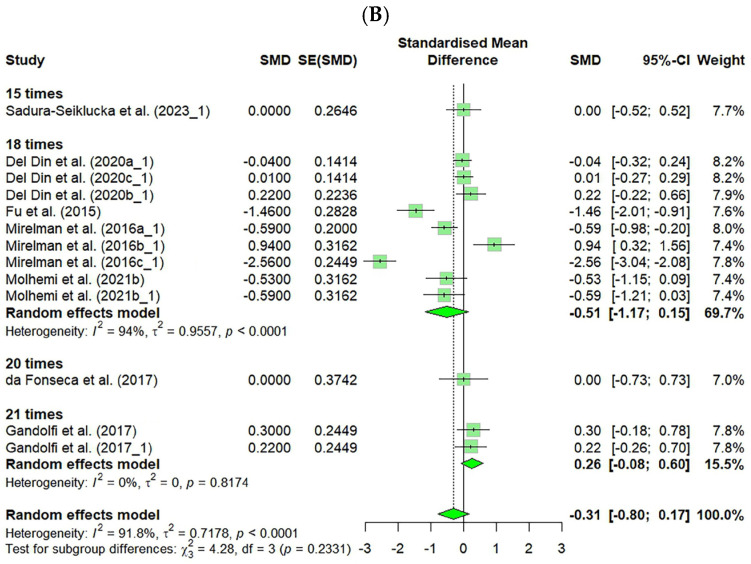
Forest plot of the effect of VR-based fall prevention intervention on the number of interventions. (**A**) Falls Efficacy Scale score. (**B**) Number of falls [15,17,19,21,31,32,34,35,36,38,39,40,41,42,43,45,46,48,50,52,53,54,55,56,58,59,60,61,62,63,65,66,67,68,69,70,71].

The results for the number of interventions showed significant improvement in the FES, with the following effect sizes: 20 times (ES = 0.70, CI: 0.34–1.05), 12 times (ES = 0.53, CI: 0.21–0.86), and 10 times (ES = 0.24, CI: 0.01–0.47). However, no significant differences were observed in the number of falls across the different intervention frequencies (Figure 4).

**Figure 5:**
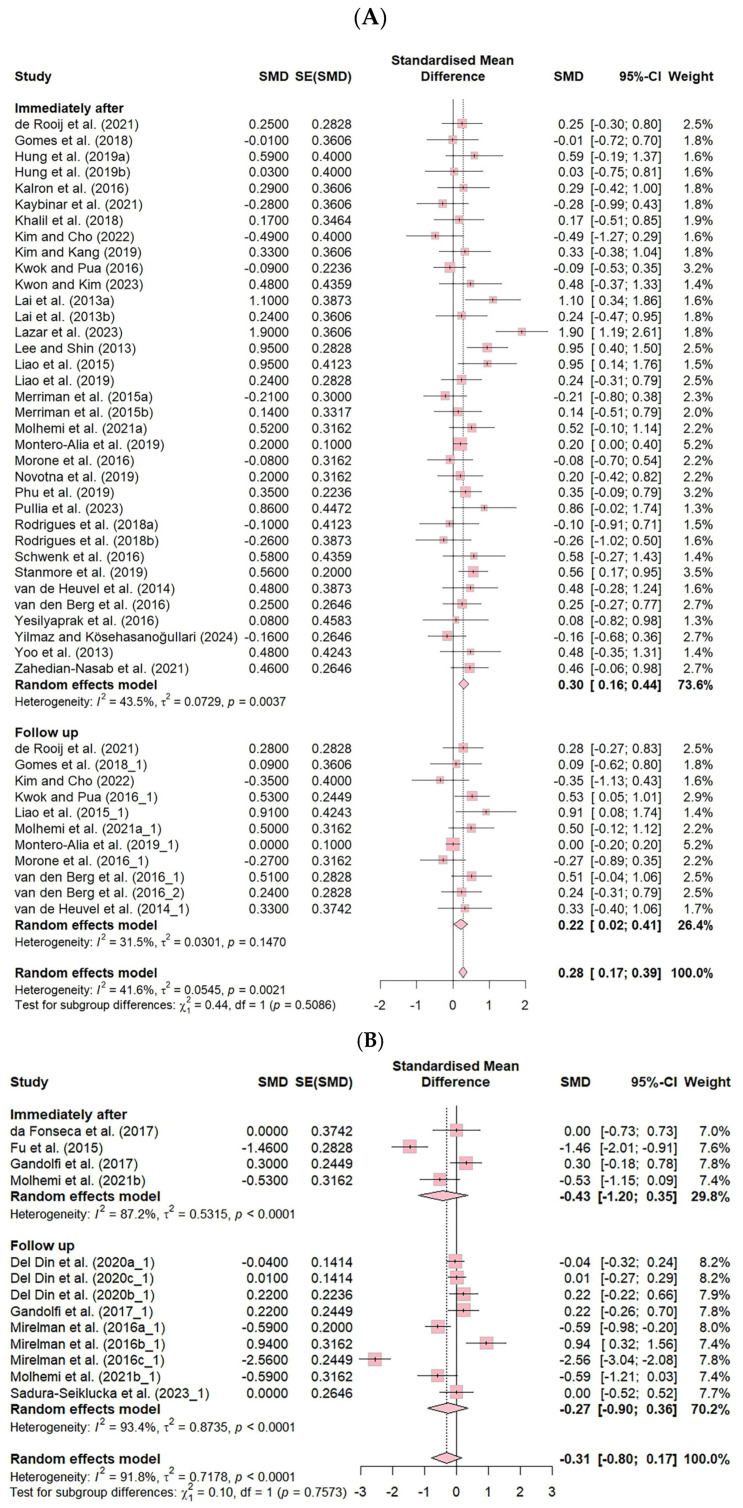
Forest plot of the effect of VR-based fall prevention intervention immediately after and at the follow-up. (**A**) Falls Efficacy Scale score. (**B**) Number of falls [15,17,19,21,31,32,34,35,36,38,39,40,41,42,43,45,46,48,50,52,53,54,55,56,58,59,60,61,62,63,65,66,67,68,69,70,71].

Additionally, the FES showed a significantly small effect size immediately after the intervention (ES = 0.30, CI: 0.16–0.44), and the follow-up studies also demonstrated a small effect size (ES = 0.22, CI: 0.02–0.41), indicating that VR-based fall prevention interventions had sustained effects. However, no significant differences were found in the number of falls across the different intervention frequencies (Figure 5).

#### 3.2.3. Results of Publication Bias and Sensitivity Analysis

**Figure 6:**
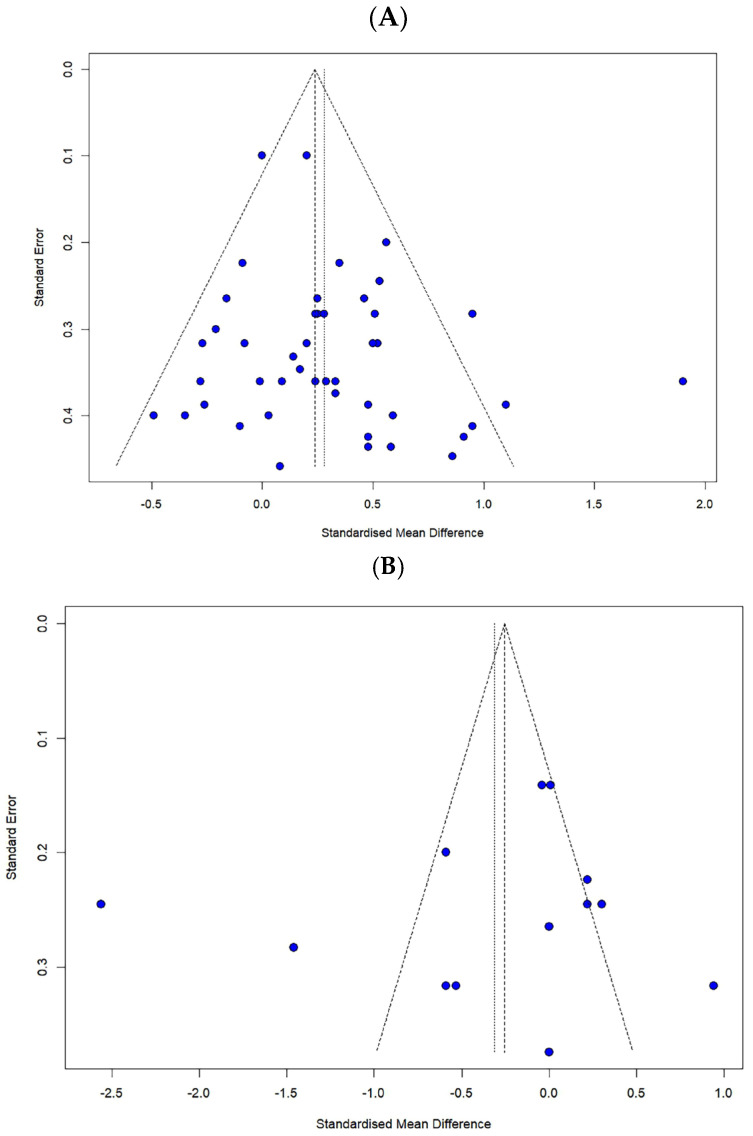
Funnel plot for (**A**) the FES and (**B**) the number of falls.

Publication bias was assessed through the visual inspection of funnel plots and statistically tested using Egger’s regression test. Egger’s test results revealed no significant publication bias for the FES (t = 1.69, *p* = 0.098) and the number of falls (t = −0.55, *p* = 0.592) (Figure 6).

Subsequently, a reanalysis using the trim-and-fill method for sensitivity analysis indicated that seven additional studies were required for the FES, while no additional studies were needed for the number of falls. Therefore, this suggests that the impact of publication bias on the overall findings is minimal.

The robustness of the findings was further examined through a sensitivity analysis using a Baujat plot and an influence graph, which are shown (Supplementary Figures S1–S4).

## 4. Discussion

The findings of this meta-analysis indicate that virtual reality (VR)-based interventions, incorporating exercise and gaming elements, are effective in improving fall-related efficacy. Although numerous meta-analyses have examined the effects of VR interventions on balance and gait, few have specifically addressed fall-related efficacy, a subjective outcome reflecting individuals’ confidence in avoiding falls. This scarcity may be because most studies on VR interventions have been conducted primarily by physicians, physical therapists, or engineers. However, in nursing science, subjective factors, such as fear of falling and self-efficacy, are recognized as key determinants of fall outcomes, highlighting the need to treat these perceptions as primary intervention targets. This is based on Bandura’s theory [72], which states that self-efficacy enhances motivation and persistence by reinforcing individuals’ confidence in performing specific tasks. In fall prevention, higher self-efficacy may promote sustained engagement in VR programs, thereby contributing to a reduced risk of falls. The present meta-analysis makes a meaningful contribution by confirming the efficacy of VR interventions in enhancing fall-related efficacy. Nevertheless, given that the observed effect sizes were modest, further research is warranted to explore strategies to maximize the effectiveness of VR interventions. For instance, incorporating mechanisms derived from self-efficacy theory [72], such as facilitating vicarious experiences by enabling multiple participants to engage in VR-based exercise together with appropriate consideration of participants’ safety, or integrating verbal persuasion techniques during exercise sessions, may enhance intervention effectiveness.

The VR program did not result in a statistically significant reduction in actual fall incidence. In addition to the multifactorial nature of fall risk factors, approximately 28–35% of older adults aged 65 years and above experience at least one fall annually, but only a subset experiences recurrent falls [73]. Consequently, large-scale studies with extended follow-up periods are necessary to assess whether interventions can effectively reduce the occurrence of falls. However, the studies included in this meta-analysis that measured the number of falls typically involved relatively small sample sizes, and only one study reported fall incidence over one year. Given that the primary goal of VR interventions is to prevent falls, future research should prioritize large-scale longitudinal studies specifically designed to monitor fall incidence over longer periods. To achieve this, it will be essential to consider the cost associated with implementing VR technology and to develop strategies that facilitate participants’ adaptation to these technologies. Moreover, most studies in this meta-analysis assessed fall frequency using retrospective self-reports, a method susceptible to recall bias and underestimation, which may have adversely affected the validity of the findings [74]. To enhance measurement accuracy, future studies should implement prospective recording approaches for fall events.

The subgroup analysis based on participants’ health conditions in the present study indicated a small effect size (0.24) for improved fall-related efficacy in older adults (k = 21), a result similar to that of Ren et al. [8], who reported a small effect size (−0.28) for reduced fear of falling in older adults (k = 13) using the FES-I. For patients with PD, the overall effect size of the VR interventions was 0.61, representing a moderate effect size or greater. Conversely, VR interventions did not have a significant effect on fall-related efficacy in patients with stroke. Given the relatively small number of studies and, consequently, the limited sample sizes for patients with MS, PD, and stroke (excluding older adult participants), additional studies are required to accurately evaluate the effects of VR interventions on fall-related efficacy within these patient populations.

Regarding the number of falls, only VR programs applied to MS patients indicated a moderate reduction in fall incidence (effect size = −0.56). Although the younger mean age of the MS participants compared to the other groups might suggest greater familiarity and engagement with VR systems, caution should be exercised in interpreting this result because it relied on only two outcomes (k = 2), both obtained from a single study with 39 participants that included immediate post-intervention and follow-up measurements.

A subgroup analysis was conducted based on the total number of VR intervention sessions, calculated by multiplying the weekly frequency (times/week) by duration (weeks). For studies using the FES as the outcome variable, the total number of intervention sessions ranged from 8 to 36. Significant improvements in efficacy were observed in the interventions provided in 10, 12, and 20 sessions. Notably, studies that delivered 12 and 20 sessions reported effect sizes equal to or greater than the medium. Based on these findings, it is recommended that VR interventions be provided for at least ten sessions, with approximately 20 sessions suggested to maximize the effectiveness.

A previous meta-analysis [8] indicated that a frequency of three sessions per week was the most effective in improving fall-related efficacy (measured by FES-I scores). Integrating these findings with the current study suggests that providing interventions three times per week over seven weeks (approximately 20 sessions) may be optimal for enhancing participants’ fall-related efficacy. Considering that subjective efficacy is enhanced through repeated successful experiences [72], it was anticipated that a greater number of intervention sessions would increase the frequency of successful experiences. However, in the current study, interventions exceeding 20 sessions did not yield significant additional effects, possibly because of the limited number of studies available. Therefore, further studies involving longer intervention durations are required.

In the studies that examined the number of falls as an outcome variable, no significant effect was observed based on the total number of VR intervention sessions. Long-term interventions may be required to demonstrate a meaningful reduction in fall incidence through VR interventions; however, the maximum total number of sessions included in this meta-analysis was 21. Thus, future studies should investigate the impact of increased total intervention sessions and extended intervention durations. Additionally, it is important to conduct research focused on developing intervention protocols that participants can implement independently, as well as on optimizing the frequency, duration, and content of interventions to sustain the benefits achieved.

A subgroup analysis was conducted based on the measurement timing, distinguishing between immediate post-intervention and follow-up assessments. The effect size for the FES measured immediately after the completion of the VR interventions was 0.30. In contrast, the effect size at follow-up was 0.22, indicating a natural decline in the effect over time. However, no clear trend of a declining effect size over time was observed, likely because of the heterogeneity in participants’ health conditions and the variety of VR intervention types. Consequently, based on the findings of this meta-analysis, it is difficult to draw definitive conclusions regarding the duration of the sustained effects of VR intervention on the FES. Future research should involve comparative studies assessing the changes in effect sizes according to follow-up timing within the same participant groups or across specific interventions.

Although fall incidence is inherently time-dependent and typically assessed over a one-year period, only one study in this meta-analysis conducted a full one-year follow-up, with the overall effect size not reaching statistical significance. This limitation likely reflects the substantial financial and practical challenges associated with implementing long-term follow-up studies. Nevertheless, given that the ultimate goal of VR interventions is to prevent falls, fall incidence should be regarded as a critical outcome measure. Therefore, future research must prioritize the accumulation of long-term follow-up studies specifically designed to evaluate fall incidence over extended periods.

This study is significant because it selected and analyzed subjective fall-related efficacy and fall incidence as outcome variables, which have not been commonly addressed in previous meta-analyses on the effects of VR interventions. Studies examining fall incidence as an outcome of VR interventions remain relatively limited. By contrast, many individual studies have used subjective fall efficacy scores as outcome measures. Furthermore, because this meta-analysis did not restrict participants to specific disease groups, it was able to include a larger number of studies than previous meta-analyses. Including a broader range of studies allowed for subgroup analyses that considered the diversity of disease characteristics, enabling comparisons of VR intervention effects across different patient groups.

This review has several limitations. First, only studies published in English and Korean were included because of language restrictions. Nevertheless, it is noteworthy that studies published in languages other than English were partially incorporated. Second, although a wide range of VR technologies and intervention modalities were identified, stratified analyses based on participants’ specific diseases or health conditions were not feasible, as the number of studies in each subgroup was insufficient for meaningful comparisons. Third, some of the included studies employed combined interventions involving both traditional exercise with VR programs; however, this review was unable to isolate the effects of VR-only interventions from those of combined approaches, making it difficult to determine the unique contributions of VR interventions to fall-related outcomes.

## 5. Conclusions

This meta-analysis found that VR interventions significantly improved subjective fall-related efficacy, with a small overall effect size. Notable improvements in fall-related efficacy were observed in older adults and participants with MS and PD. At least 10 intervention sessions were necessary to achieve meaningful improvements in fall-related efficacy, with 20 sessions resulting in a medium or greater effect size. However, the VR intervention did not demonstrate a definitive effect in reducing the number of falls. Given the importance of fall events in evaluating VR intervention outcomes, future research should prioritize large-scale, longitudinal studies with extended follow-up periods. Studies should adopt fall incidence as a primary outcome and use prospective monitoring methods to ensure accurate and unbiased data collection.

## Figures and Tables

**Table 1:** Characteristics of the included studies.

No.	Author (Year)	Design	Country	Age [Mean ± SD or Median (IQR)] (IG/CG(s))	Disease	Sample Size (IG/CG(s))	Single Session Time (min) Weekly Frequency; Total Duration F/U Time	Technology	Intervention	Control	Outcome Measure
1	Bekkers et al. (2020) [20]	RCT	UK	71.06 ± 6.3 70.86 ± 6.0	Parkinson’s disease	121 (62/59)	45 min 3/w; 6 w F/U after 6 m	V-time	Treadmill training, VR	Treadmill training	FES-I
2	Collado-Mateo et al. (2017) [47]	RCT	Spain	52.43 ± 9.83 52.58 ± 9.42	Other (fibromyalgia)	76 (41/35)	60 min 2/w; 8 w	Virtual EX-FM (Microsoft Kinect R)	Warm-up + postural control and coordination + mobility skills, balance, and coordination	Usual activity	FoF
3	da Fonseca et al. (2017) * [48]	RCT	Brazil	53.8 ± 6.3 50.9 ± 10.9	Stroke	27 (14/13)	60 min 2/w; 10 w	Nintendo Wii	Virtual rehabilitation	Conventional training	Number of falls
4	de Rooij et al. (2021) * [19]	RCT	The Netherlands	65 (57–70) 61 (53–71)	Stroke	52 (28/24)	30 min 2/w; 6 w F/U after 3 m	GRAIL: Motek force Link (Treadmill)	VR gait training	Non-VR gait training	FES-I
5	Del Din et al. (2020) * [34]	RCT	USA	[75.93 ± 6.22/ 78.03 ± 6.21/ 71.68 ± 6.43] **	Other (healthy/MCI/PD)	275 (109/38/128)	40 min 3/w; 6 w F/U after 6 m	V-time	Treadmill training, VR	Treadmill training	Number of falls FRA
6	Duque et al. (2013) [44]	RCT	Australia	79.3 ± 10 75.0 ± 8	Healthy	60 (30/30)	30 min 2/w; 6 w	Balance rehabilitation unit	Visual–vestibular training + postural training	Usual care (Otago Exercise Program)	Number of falls FoF
7	Eftekharsadat et al. (2015) [49]	RCT	Iran	33.4 ± 8.1 37.0 ± 8.3	Multiple sclerosis	30 (15/15)	20 min 2/w; 12 w	Biodex Balance System	VR-based balance training	No intervention	FRI
8	Fu et al. (2015) * [45]	RCT	Hong Kong	82.4 ± 3.8 82.3 ± 4.3	Healthy	60 (30/30)	60 min 3/w; 6 w F/U after 12 m	Nintendo Wii Balance Board	Exergaming balance training	Conventional balance training	Number of falls
9	Gandolfi et al. (2017) * [46]	RCT	Italy	67.45 ± 7.18 69.84 ± 9.41	Parkinson’s disease	76 (38/38)	50 min 3/w; 7 w F/U after 12 m	Nintendo Wii Fit system	Home VR telerehabilitation balance training	Clinic sensory integration balance training	Number of falls
10	Gomes et al. (2018) * [50]	RCT	Brazil	83 ± 5.87 85 ± 6.19	Healthy	30 (15/15)	50 min 2/w; 7 w F/U after 30 days	Nintendo Wii Fit Plus	Nintendo Wii Fit Plus games	Booklet information and illustrations outlining the benefits and risks of physical activity	FES-I
11	Htut et al. (2018) [51]	RCT	Thailand	75.8 ± 4.89 [75.9 ± 5.65 75.6 ± 5.33 76.0 ± 5.22]	Healthy	42 (21/21)	30 min 3/w; 8 w	Xbox 360 with Microsoft’s Kinet (Flextronics, Wistron, Celestica, Foxconn)	VR-based exercise	Physical ex Brain ex No intervention	FES-I
12	Hung et al. (2019) * [52]	Randomized crossover design	Taipei	IG first: 71.0 ± 1.22 CG first: 66.5 ± 2.10	Other (diabetes mellitus)	24 (12/12)	30 min 3/w; 6 w	Xavix PORT (Shinsedai Co Ltd.)	IVGB balance training program	No intervention	MFES
13	Kalron et al. (2016) * [53]	RCT	Israel	47.3 ± 9.6 43.9 ± 10.6	Multiple sclerosis	30 (15/15)	30 min 2/w; 6 w	CAREN (Motek)	Motion platform projection	Conventional exercise program	FES-I
14	Kanyilmaz et al. (2022) [18]	RCT	Turkey	70 ± 6.00 70 ± 5.00	Other (dizziness)	26 (13/13)	30 min 5/w; 3 w F/U after 6 m	C-Mill (Treadmill)	VR-based vestibular rehabilitation	Vestibular rehabilitation	FES-I FRI
15	Kayabinar et al. (2021) * [15]	RCT	Turkey	58.8 ± 5.03 57.06 ± 6.75	Stroke	30 (15/15)	30 min 3/w; 6 w	Robot Gait VR	Preparation, gait, VR game	Preparation, gait	FES-I
16	Khalil et al. (2018) * [54]	RCT	Jordan	39.88 ± 12.85 34.87 ± 8.98	Multiple sclerosis	32 (16/16)	4 sets of 1 min per Ex 3/w; 6 w	Nintendo Wii Balance Board	Wii Balance Board exercise, 12 sessions + 6 sessions at home	Conventional home balance training	FES-I
17	Kim and Cho (2022) * [55]	RCT	Korea	78.75 ± 10.15 80.75 ± 6.03	Healthy	24 (12/12)	30 min 3/w; 6 w F/U after 8 w	Wii Fit game training	Balance training (Wii Fit games)	No intervention	FES
18	Kim and Kang (2019) * [56]	RCT	KOREA	72.13 ± 7.70 76.00 ± 9.52	Parkinson’s disease	30 (15/15)	30 min 5/w; 4 w	Interactive Rehabilitation and Exercise System	VR-based exercise program	Conventional physical therapy	FES
19	Ku et al. (2018) [57]	RCT	KOREA	64.7 ± 7.27 65.0 ± 4.77	Healthy	34 (18/16)	30 min 3/w; 4 w	Three-dimensional interactive augmented reality system (3D-ARS) using Microsoft Kinect sensor	Balloon/cave/rhythm game	Conventional physical fitness program	FRI
20	Kwok and Pua (2016) * [38]	RCT	Singapore	70.5 ± 6.7 69.8 ± 7.5	Healthy	73 (37/36)	60 min 1/w; 12 w F/U after 24 w	Nintendo Wii Active gaming exercise with Wii Balance Board	Wii exercise program + resistance band	Gym exercise class	Number of falls, Number of fallers, MFES
21	Kwon and Kim (2023) * [39]	RCT	KOREA	69.00 ± 7.90 68.10 ± 8.33	Stroke	20 (10/10)	30 min 5/w; 4 w	Theta trainer Balo + Theta-Soft	Dual task program + conventional occupational therapy	Conventional occupational therapy	FES-I
22	Lai et al. (2013) * [58]	Randomized crossover design	Taiwan	IG first: 70.6 ± 3.5 CG first: 74.5 ± 4.7	Healthy	30 (IG first 15 CG first 15)	30 min 3/w; 6 w	Xavix Step System	IVGB exercise (Interactive Video game-based exercise)	No intervention	MFES
23	Lazar et al. (2023) * [32]	NRCT	India	69.44 ± 6.66 66.33 ± 6.51	Healthy	44 (22/22)	30 min 3/w; 4 w	No information	Immersive VR training	Conventional balance training	FES-I
24	Lee and Shin (2013) * [40]	RCT	Korea	73.78 ± 4.77 74.29 ± 5.20	Other (diabetes mellitus)	55 (27/28)	50 min 2/w; 10 w	Video gaming PlayStation 2	Warm-up exercise VR exercise cool down exercise	Diabetes education	MFES
25	Levy et al. (2016) [30]	RCT	France	72.4 ± 12.25 68.65 ± 19.05	Healthy	16 (9/7)	40 min 1/w; 12 w	Eye Toy interface for PlayStation 2	VR exposure therapy	Conventional training	FoF
26	Liao et al. (2015) * [59]	RCT	Taiwan	67.3 ± 7.1 64.6 ± 8.6	Parkinson’s disease	24 (12/12)	45 min 2/w; 6 w F/U after 30 days	Nintendo Wii Fit Plus gaming system and Wii Fit Balance Board	VR-based Wii Fit exercise	Traditional Ex/ education	FES-I
27	Liao et al. (2019) * [60]	RCT	Taiwan	79.6 ± 8.5 84.1 ± 5.5	Healthy	52 (27/25)	60 min 3/w; 12 w	Xbox Kinect (exergame)	Kinect-based game (tai-chi, resistance and aerobic, balance games)	Conventional exercise (resistance and aerobic, balance exercise)	FES-I
28	Merriman et al. (2015) * [61]	NRCT	Ireland	[74.90 ± 8.97/ 74.06 ± 6.66 ***] [74.33 ± 11.09/73.41 ± 7.00 ***]	Healthy	76 (38/38)	30 min 2/w; 5 w	Nintendo Wii Balance Board	Balance training (custom-designed games)	No intervention	FES
29	Mirelman et al. (2016) * [35]	RCT	Belgium Israel, Italy the Netherlands, UK	74.2 ± 6.9 73.3 ± 6.4	Other (healthy/MCI/PD)	282 (146/136)	45 min 3/w; 6 w F/U after 6 m	V-time (Microsoft Kinect)	Treadmill training, VR	Treadmill training	Number of falls, Number of fallers
30	Molhemi et al. (2021) * [21]	RCT	Iran	36.8 ± 8.4 41.6 ± 8.4	Multiple sclerosis	39 (19/20)	30 min 3/w; 6 w F/U after 3 m	Xbox 360 with Microsoft’s Kinet	VR balance training	Conventional balance training	Number of falls Number of fallers FES-I
31	Montero-Alia et al. (2019) * [31]	NRCT	Spain	75.1 (72.6–78.7) 75.4 (72.7–78.6)	Healthy	630 (274/356)	30 min 2/w; 3 m F/U after 1 yr	Nintendo Wii Fit console and the WBB	Balance training (Wii Fit games)	Usual care	SF-FES-I
32	Morone et al. (2016) * [41]	RCT	Italy	67.80 ± 2.98 70.05 ± 4.93	Other (bone loss condition)	38	60 min 2/w; 8 w F/U after 3 m	Wii Fit program	Exercise + Wii balance games	Rehabilitation program for preventing and treating women with postmenopausal osteoporosis	SF-FES-I
33	Novotna et al. (2019) * [62]	RCT	Czech	39.39 ± 9.68 42.56 ± 10.63	Multiple sclerosis	39 (23/16)	15 min 7/w; 4 w F/U after 4 w	Homebalance^®^ system	Home-based balance training	No intervention	FES-I
34	Pelosin et al. (2019) [33]	RCT	Italy	73.2 ± 3.6 71.9 ± 4.1	Parkinson’s disease	39 (17/22)	45 min 3/w; 6 w	No information	Treadmill training, VR	Treadmill training	Number of falls
35	Phu et al. (2019) * [63]	NRCT	Australia	79 (74–84) 79 (72–82)	Healthy	113 (63/50)	60 min 2/w; 8 w	Balance rehabilitation unit	VR balance training (postural training + rehabilitation)	Education regarding fall risk	FES-I
36	Pullia et al. (2023) * [36]	RCT	Italy	64.5 ± 10.84 65.5 ± 10.36	Parkinson’s disease	20 (10/10)	45 min 4/w; 5 w	C-Mill (Treadmill)	Innovative gait training using C-Mill	Conventional training	FES-I
37	Rebêlo et al. (2021) [64]	RCT	Brazil	69.25 ± 5.27 71.41 ± 5.94	Other (balance disorders)	37 (20/17)	50 min 2/w; 8 w F/U after 2 m	Oculus rift	VR-based balance training	Balance training through exercise	FES-I
38	Rieger et al. (2023) [37]	RCT	The Netherlands	75.5 ± 5.40 73.9 ± 5.94	Healthy	70 (35/35)	30 min 2/w; 4 w F/U after 6 m	C-Mill (Treadmill)	VR-based training	Conventional training	Number of falls, Number of fallers, FES-I
39	Rodrigues et al. (2018) * [65]	NRCT	Brazil	[68.9 ± 3.3/ 69.8 ± 4.3 **†**] [68.7 ± 4.8/ 73.6 ± 5.4 **†**]	Healthy	45 (22/25)	40 min 3/w; 12 w	Dance Central game for Xbox 360 + Kinect motion sensor	Pop dance exergaming in group sessions	No intervention	Number of fall FES-I
40	Sadura-Seiklucka et al. (2023) * [42]	RCT	Poland	63 ± 8 65 ± 8	Other (osteoarthritis)	57 (28/29)	30 min 5/w; 3 w F/U after 3 m	Virtual balance training on force plate (Pro-Med)	Comprehensive rehabilitation + force plate training	Comprehensive rehabilitation + conventional training	Number of falls
41	Schwenk et al. (2016) * [66]	RCT	USA	77.8 ± 6.9 79.0 ± 10.4	Other (MCI)	20 (11/9)	45 min 2/w; 4 w	LegSys (BioSensics LLC)	Point-to-point reaching tasks + virtual obstacle-crossing tasks	No intervention	SF-FES-I
42	Silva et al. (2024) [16]	RCT	Portugal	61.73 ± 6.54 67.46 ± 8.59	Parkinson’s disease	30 (15/15)	60 min 3/w; 12 w	HMD (HTC Vive^TM^ Pro	Exergame BOX VR combination (physiotherapy + VR)	Physiotherapy	Number of falls
43	Stanmore et al. (2019) * [17]	cluster RCT	UK	77.9 ± 8.9 77.8 ± 10.2	Healthy	92 (49/43)	30 min 3/w; 12 w, F/U after 3 m (only fall diary)	Xbox 360 with Microsoft’s Kinet	Exergame + standard care (Otago-based exercise)	Standard care (Otago-based exercise) only	FES-I FRAT
44	van den Berg et al. (2016) * [67]	RCT	Australia	78 ± 10 82 ± 13	Other (rehabilitation patient)	56 (27/29)	30 min 5/w; 2 w F/U after 6 w and after 12 w	Nintendo Wii Fit, Xbox 360 with Microsoft’s Kinet, HUMAC balance system, Fitbit Zip	Video- and computer-based interactive exercise	Usual care	FES
45	van den Heuvel et al. (2014) * [68]	RCT	The Netherlands	66.3 ± 6.39 68.8 ± 9.68	Parkinson’s disease	27 (15/12)	60 min 2/w; 5 w F/U after 6 w	Workstation with force plate and/or inertial sensor	Visual feedback-based balance training	Conventional balance training	FES
46	Yesilyaprak et al. (2016) * [69]	RCT	Turkey	70.1 ± 4.00 73.1 ± 4.50	Healthy	18 (7/11)	30–60 min 3/w; 6 w F/U after 6 m	BTS NIRVABA VR Interactive System	VR-based balance exercise	Conventional balance exercise	FES-I
47	Yilmaz and Kösehasanoğulları (2024) * [70]	RCT	Turkey	67 ± 10.64 68 ± 9.06	Other (osteoporosis)	60 (30/30)	45 min 3/w; 12 w	Wii Fit game training	Balance training (Wii Fit games)	Home exercise group	FES
48	Yoo et al. (2013) * [43]	RCT	Korea	72.90 ± 3.41 75.64 ± 5.57	Healthy	21 (10/11)	60 min 3/w; 12 w	I-visor FX6011	VR-based Otago exercise	Otago exercise	FES-I
49	Zahedian-Nasab et al. (2021) * [71]	RCT	Iran	69.67 ± 7.73 72 ± 7.81	Healthy	60 (30/30)	30–60 min 2/w; 6 w	Xbox Kinect (exergame)	Ski, penalty and goalkeeper, darts	Routine program	SF-FES

*: included in the meta-analysis; **: ages of healthy, MCI, and PD groups; ***: fall-prone group; **†**: faller group. IG: intervention group, CG(s): comparison group(s); F/U: follow-up; min: minute; w: week; m: mon; yr: year; MCI: mild cognitive disorder; PD: Parkinson’s disease. MCI: mild cognitive impairment; Ex: exercise; FES-I = Fall Efficacy Scale-International; FES = Fall Efficacy Scale; FoF = Fear of Fall; FRA = Falls Rate to Activity; FRI = Fall Risk Index; MFES = Modified Fall Efficacy Scale; SF = FES-I = Short-Form Fall Efficacy Scale-International; FRAT = Fall Risk Assessment Tool.

## Data Availability

The data underpinning this study are available in this article and in its Supplementary Materials.

## Supplementary Materials

The following supporting information can be downloaded at https://www.mdpi.com/article/10.3390/healthcare13151845/s1. Supplementary Table S1. Search strategies; Supplementary Table S2. Quality of evidence by GRADE for the FES and the number of falls; Supplementary Figure S1. Baujat plot for the FES; Supplementary Figure S2. Baujat plot for the number of falls; Supplementary Figure S3. Influence graph for the FES; Supplementary Figure S4. Influence graph for the number of falls.
