# Supplementary material for: Effects of Virtual Reality Based on Fall Prevention Intervention: A Systematic Review and Meta-Analysis
Source: Healthcare (Basel). 2025 Jul 29;13(15):1845. doi: 10.3390/healthcare13151845 (PMC12346508; doi:10.3390/healthcare13151845)
Supplement: Supplementary file 1 [file healthcare-13-01845-s001.zip › healthcare-3688535-supplementary.pdf]

Effects of Virtual Reality Based on Fall Prevention Intervention: A Systematic Review and Meta-analysis

Supplementary Table S 1: Search Strategies

No	Databases		Searches	Results
1	EMBASE	#1	Virtual Reality [EMTREE] OR Virtual reality [All field]	7,099
		#2	Falling [EMTREE] OR fall OR fall prevention [All field]	32,599
		#3	#1 AND #2	576
2	Cochrane Library	#1	Virtual Reality OR Virtual realit*	8,003
		#2	fall OR fall* OR fall* prevention	31,628
		#3	Virtual Realit* AND fall*	386
3	CINAHL	#1	Virtual Reality OR Virtual reality	195,965
		#2	fall OR fall prevention	428,894
		#3	#1 AND #2	867
4	PubMed	#1	Virtual Reality [MesH] OR Virtual Realt* [All field]	815
		#2	Accidental falls [MesH] OR fall* OR fall* prevention [All field]	4,408
		#3	#1 AND #2	31
5	RISS	#1	Virtual Reality	3,031
		#2	fall	611
		#3	Virtual Reality AND fall	17
6	DBpia	#1	Virtual Reality	2,747
		#2	fall	513
		#3	Virtual Reality AND fall	13
7	KISS	#1	Virtual Reality	2,969
		#2	fall	578
		#3	Virtual Reality AND fall	9
8	KoreaMed	#1	Virtual Reality	98
		#2	fall	1,142
		#3	Virtual Reality AND fall	1

Supplementary Table S 2: Quality of evidence by GRADE for FES and Number of falls

Summary findings		Certainty of evidence based on the GRADE approach					Level of evidence
Outcome	Number of studies	Risk of bias	Inconsistency	Indirectness	Imprecision	Publication bias	
Fall efficacy scale	36	Serious (-1)	No serious limitation	No serious limitation	No serious limitation	No serious limitation	Moderate
Number of falls	13	Serious (-1)	Very serious (-2)	No serious limitation	No serious limitation	No serious limitation	Very low

GRADE: Grading of Recommendations Assessment, Development and Evaluation

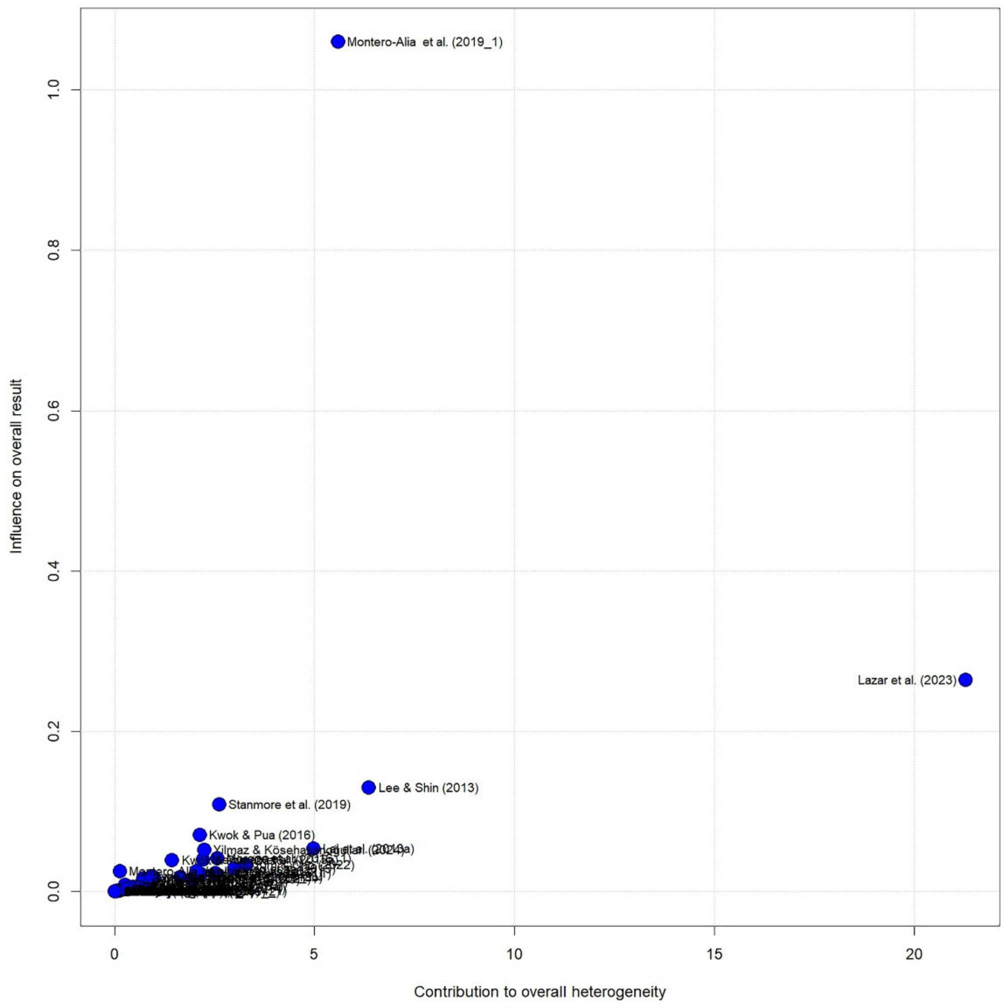

Supplementary Figure S 1: Baujat plot for FES

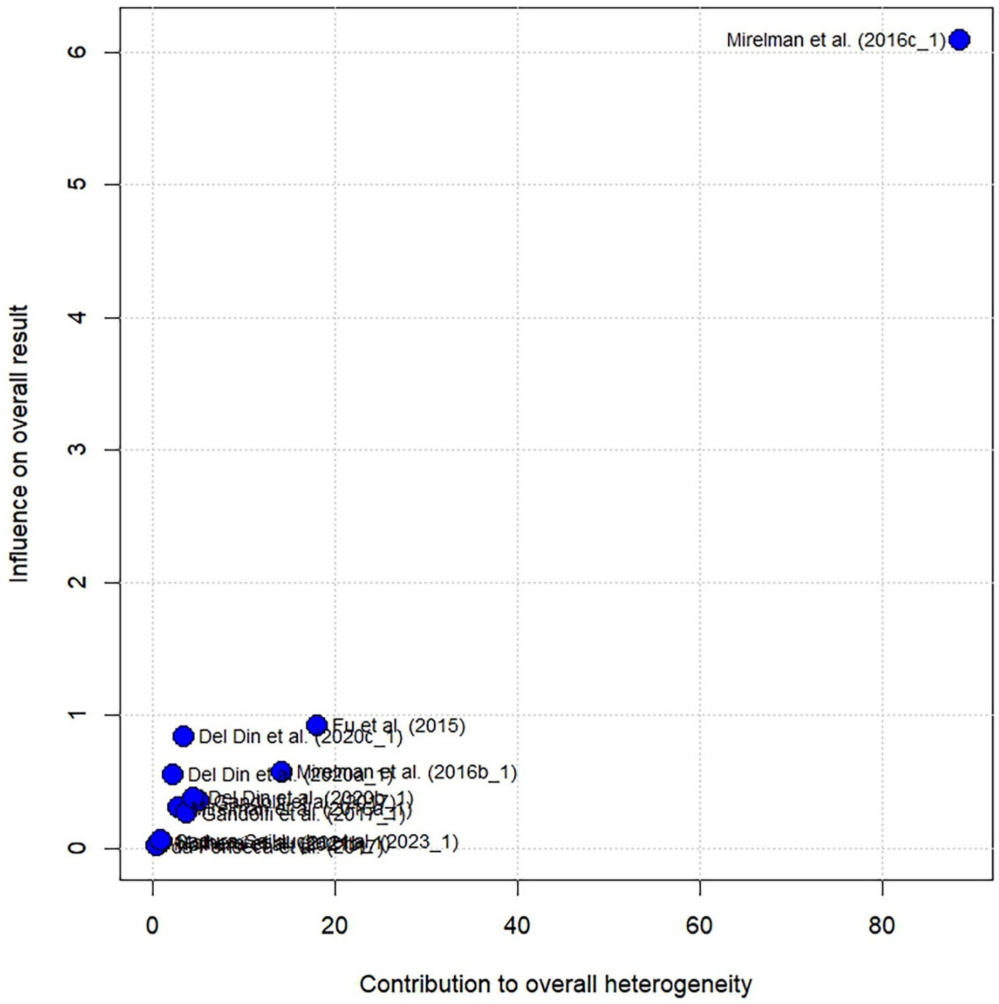

Supplementary Figure S 2: Baujat plot for number of falls

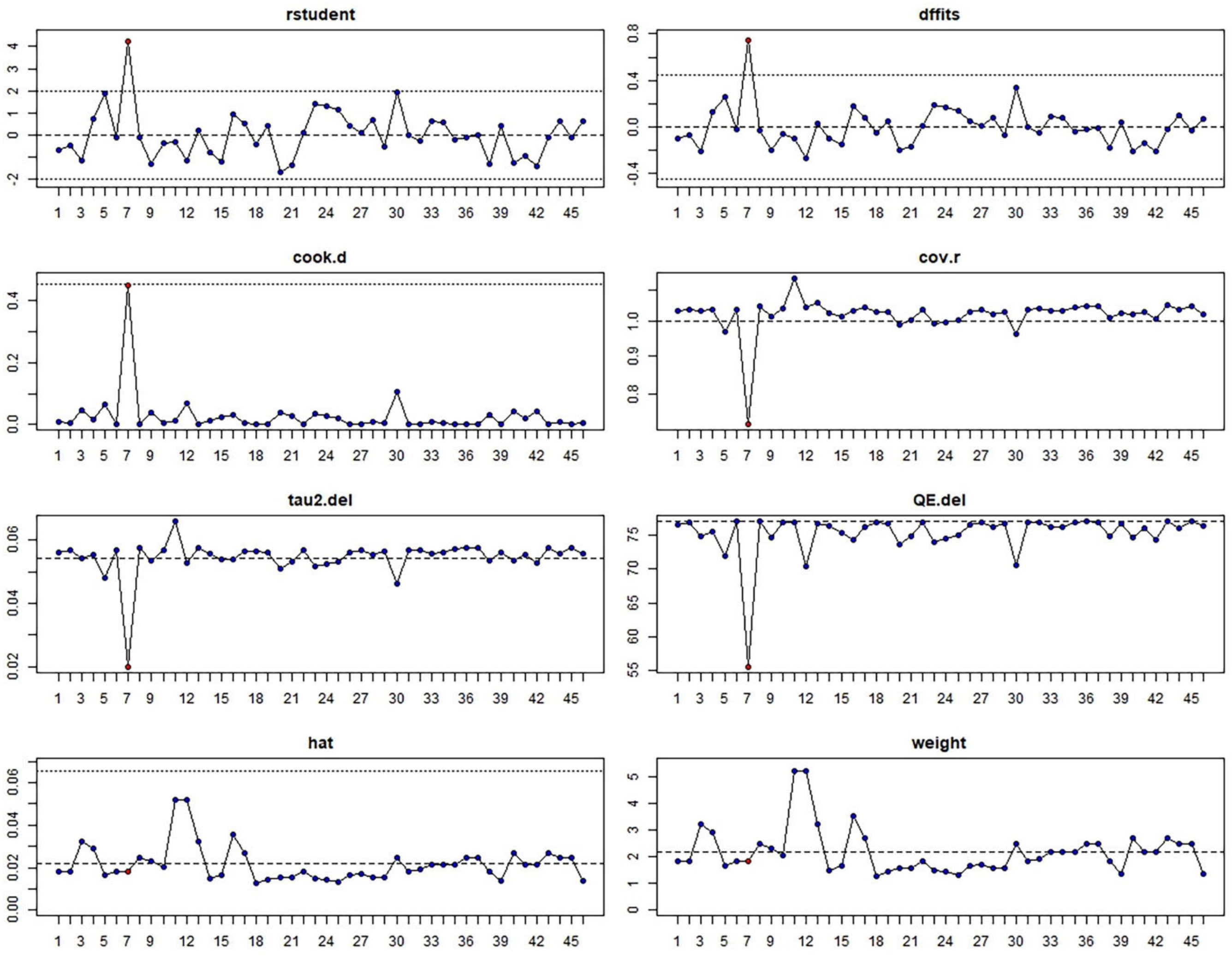

Supplementary Figure S 3: Influence graph for FES

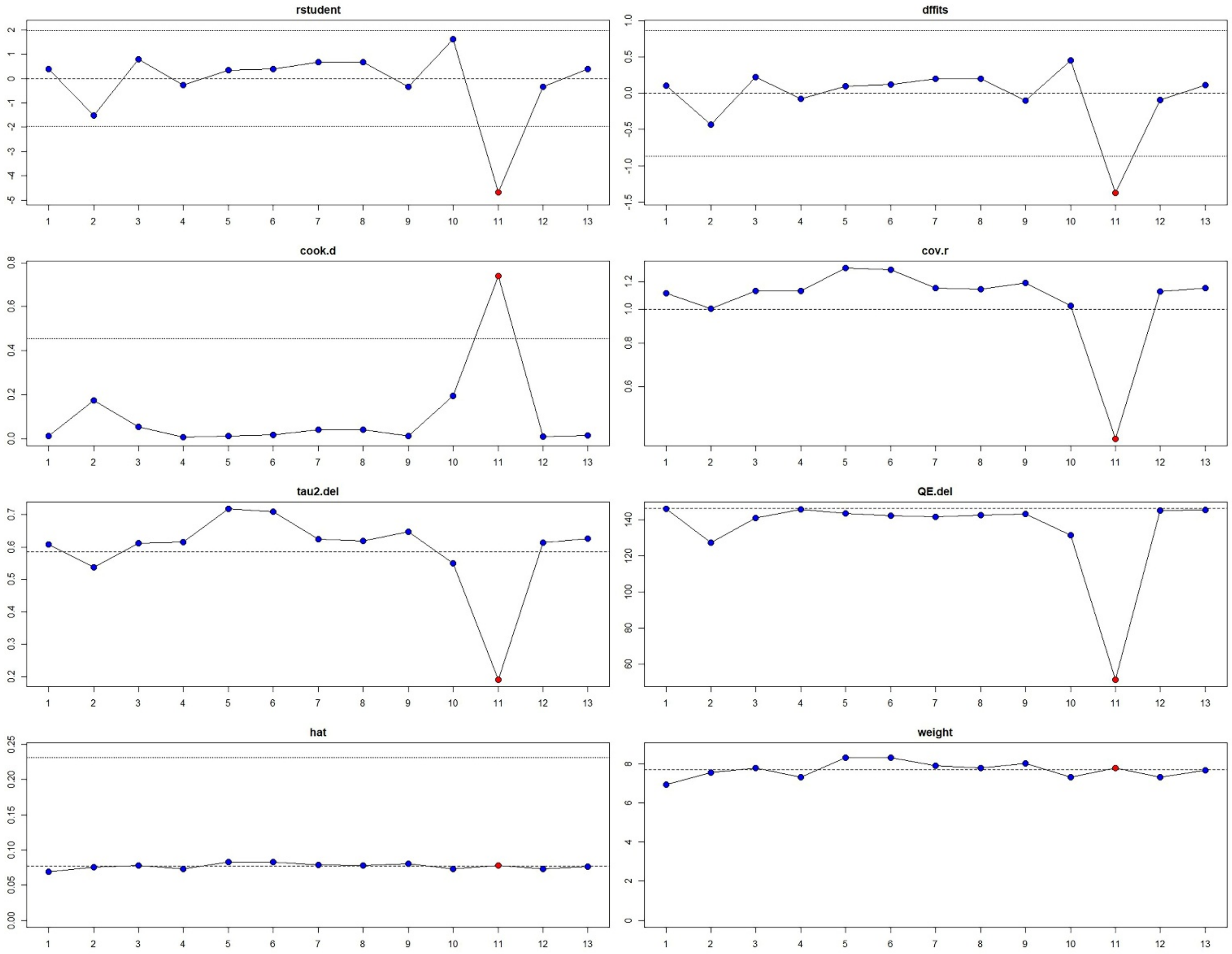

Supplementary Figure S 4: Influence graph for number of falls
